# Targeted Isolation of Antibiofilm Compounds from Halophytic Endophyte *Bacillus velezensis* 7NPB-3B Using LC-HR-MS-Based Metabolomics

**DOI:** 10.3390/microorganisms12020413

**Published:** 2024-02-19

**Authors:** Sanju Singh, Elizabeth Nwagwu, Louise Young, Pankaj Kumar, Pramod B. Shinde, RuAngelie Edrada-Ebel

**Affiliations:** 1 Natural Products & Green Chemistry Division, CSIR-Central Salt and Marine Chemicals Research Institute (CSIR-CSMCRI), Council of Scientific and Industrial Research (CSIR), Bhavnagar 364002, India; sanju.singh@strath.ac.uk (S.S.); pawar.p08@gmail.com (P.K.); 2 Academy of Scientific and Innovative Research (AcSIR), Ghaziabad 201002, India; 3 Strathclyde Institute of Pharmacy and Biomedical Sciences, University of Strathclyde, The John Arbuthnott Building, 161 Cathedral Street, Glasgow G4 0RE, UK; elizabeth.nwagwu@strath.ac.uk (E.N.); louise.c.young@strath.ac.uk (L.Y.)

**Keywords:** antibiofilm, dereplication, endophytes, halophyte, metabolite profiling, multivariate analysis

## Abstract

The discovery of new natural products has become more challenging because of the re-isolation of compounds and the lack of new sources. Microbes dwelling in extreme conditions of high salinity and temperature are huge prospects for interesting natural metabolites. In this study, the endophytic bacteria *Bacillus velezensis* 7NPB-3B isolated from the halophyte *Salicornia brachiata* was screened for its biofilm inhibition against methicillin-resistant *Staphylococcus aureus* (MRSA). The fractionation of the crude extract was guided by bioassay and LC-HRMS-based metabolomics using multivariate analysis. The 37 fractions obtained by high-throughput chromatography were dereplicated using an in-house MS-Excel macro coupled with the Dictionary of Natural Products database. Successive bioactivity-guided separation yielded one novel compound (**1**), a diketopiperazine (*m*/*z* 469.258 [M − H]^−^) with an attached saturated decanoic acid chain, and four known compounds (**2**–**5**). The compounds were identified based on 1D- and 2D-NMR and mass spectrometry. Compounds **1** and **5** exhibited excellent biofilm inhibition properties of >90% against the MRSA pathogen at minimum inhibition concentrations of 25 and 35 µg/mL, respectively. The investigation resulted in the isolation of a novel diketopiperazine from a bacterial endophyte of an untapped plant using an omics approach.

## 1. Introduction

Bacterial biofilms cause serious global health concerns contributing to persistent chronic infections. Biofilms provide a shield for pathogenic bacteria against the host immune system by inhibiting phagocyte function and suppressing complement system activation, thereby enhancing bacterial resistance against conventional antibiotics by around 1000-fold [1,2]. Multidrug-resistant bacteria like *Staphylococcus aureus* are responsible for nosocomial and community-acquired infections globally causing numerous diseases, such as food poisoning, skin infections, pneumonia, and septicemia. Biofilm-forming staphylococci for instance *S. epidermis* and *S. aureus* cause biomaterial-associated infections [3]. Hence, it is imperative to develop new means of eradication to combat resistant pathogens [4].

Fermentation bioprocessing technology in microbial systems has elevated their use in the isolation of commercially important metabolites. There lies a gap in research and investigation of beneficial compounds from extreme environments, such as marine, desert, and marshlands. Endophytic bacteria from halophytes hold a vast antimicrobial potential against pathogenic fungi and bacteria [5]. Among various biocontrol agents, *Bacillus* sp. has demonstrated strong abilities to restrict microbial pathogens of various niches by the production of hydrolytic enzymes, peptides, and polyketides [6]. *B. velezensis* is a recently classified species of the genus *Bacillus* [7,8] and is reported to exhibit antifungal activity, plant growth promotion properties, dye detoxification, and keratinolytic, dehairing, and proteolytic actions. The versatility extends to the production of enzymes such as protease and cellulase, bioremediation of organophosphorus pesticides, and generation of probiotics for animal feeds [7,9,10]. We hypothesized that investigation of endophytic *B. velezensis* from a halophyte growing in extreme conditions can lead to the isolation of some interesting metabolites. Compounds such as surfactin, fengycin, bacilysin, bacillibactin, bacillaene, macrolactin, and difficidin are reported from varied *B. velezensis* strains [11,12,13]. An antimicrobial peptide *YS12* (mwt. 3348 Da) was reported from *B. velezensis* CBSYS_12_ that exhibited antibiofilm properties against bacterial pathogens [14]. The presence of secondary metabolite biosurfactants such as surfactins also contributes to the antibiofilm capacity of *B. velezensis* strains [15,16,17].

The discovery of new potential bioactive compounds from natural niches is challenging because of the re-isolation of compounds. Metabolite profiling of crude extracts in amalgamation with dereplication using natural product databases can facilitate rapid and high-throughput assessment of metabolites. Dereplication benefits by screening out the known metabolites in the crude extract to guide the isolation procedure for novel metabolites [18]. In this study, we investigate an endophyte *B. velezensis* 7NPB-3B isolated from the halophytic plant *Salicornia brachiata* for antibiofilm compounds using metabolomics in concatenation with bioassay-guided separation. We performed LC-MS-based metabolomics of the crude extract and fractions using multivariate analysis, i.e., PCA and OPLS-DA to determine the significant metabolites responsible for the bioactivity. Putative identification of the metabolites was carried out employing the Dictionary of Natural Products (DNP) database. The study structurally characterized and identified the bioactive compounds capable of effectively controlling MRSA biofilms by using NMR spectroscopy and mass spectrometry techniques.

## 2. Results and Discussion

**Figure 1:**
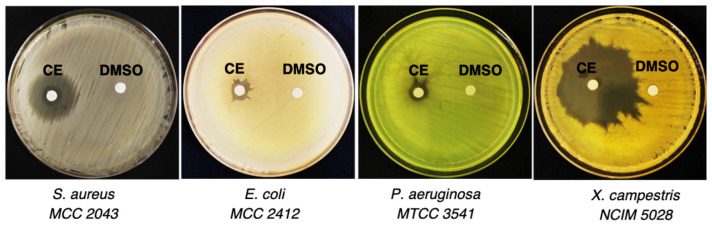
Preliminary antimicrobial screening of *B. velezensis* 7NPB-3B crude extract (CE) against four different pathogens using disk diffusion assay at a concentration of 1 mg/mL and loading volume of 10 µL keeping DMSO as vehicle control.

Recently, our laboratory isolated around 350 bacterial endophytes from the halophyte *Salicornia brachiata* wildly distributed along the coast of Gujarat, India. The endophytic strain of interest, 7NPB-3B was identified as *Bacillus velezensis* with the GenBank accession number MT645763 based on its 16S rRNA sequence and phylogenetic analysis performed using MEGA X [5]. *B. velezensis* 7NPB-3B displayed a broad antibacterial potential against pathogenic bacteria, such as *S. aureus* MCC 2043, *E. coli* MCC 2412, *P. aeruginosa* MTCC 3541, and *X. campestris* NCIM 5028 (Figure 1). *B. velezensis* species is a recently identified species under the genus *Bacillus* [8]; therefore, it is important to explore its bioactive potential to determine a distinguishing factor with respect to its contemporaries. Therefore, bioactivity-guided isolation of antibiofilm metabolites from the strain was attempted using MRSA as the biofilm-forming agent.

**Figure 2:**
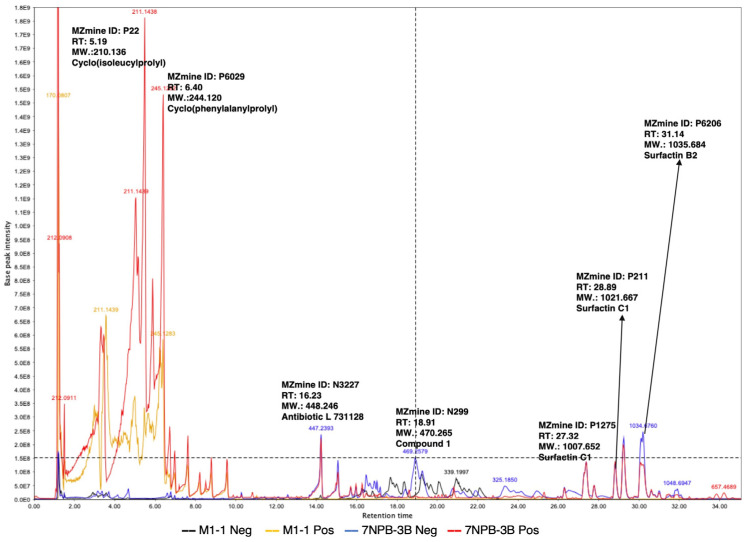
LC-MS total ion chromatogram (TIC) of *B. velezensis* 7NPB-3B crude extract in positive (—) as well as negative mode (—) compared with media control in positive (—) and negative mode (—). The labeled ion peaks represent some of the significant metabolites.

For the extraction of bioactive metabolites, a large-scale culture of *B. velezensis* 7NPB-3B was performed and the culture broth was extracted with ethyl acetate resulting in 13.9 g crude extract. The crude extract was partitioned between methanol and hexane to remove the non-polar or possible fatty acid constituents. The total ion chromatogram (TIC) of the crude extract of *B. velezensis* 7NPB-3B (Figure 2) when dereplicated using the DNP database showed the existence of known and unknown compounds present in the extract (Table 1). Some of the putatively identified compounds have been previously isolated from microbes including the genus *Bacillus*. Characteristic metabolites known from the genus were annotated such as a series of more non-polar surfactin congeners with molecular weights at ca. 1000 Da eluting after 25 min. Some ion mass peaks were found to have “No Hit”. However, their peak areas and predicted formulas were also listed. On the other hand, four of the dereplicated features were plant metabolites, which could indicate that referred ion peaks may instead belong to an unreported or unknown metabolite yet to be discovered. The dereplication study revealed that the bacterial extract possesses a wide arena of compounds such as lipopeptides, diketopiperazines, polyketides, quinones, amino alcohols, and macrolides isolated from microbial sources either fungi or bacteria. The number of probable hits was filtered by biological sources that were closer to our strain of interest, i.e., *B. velezensis* 7NPB-3B.

**Scheme 1:**
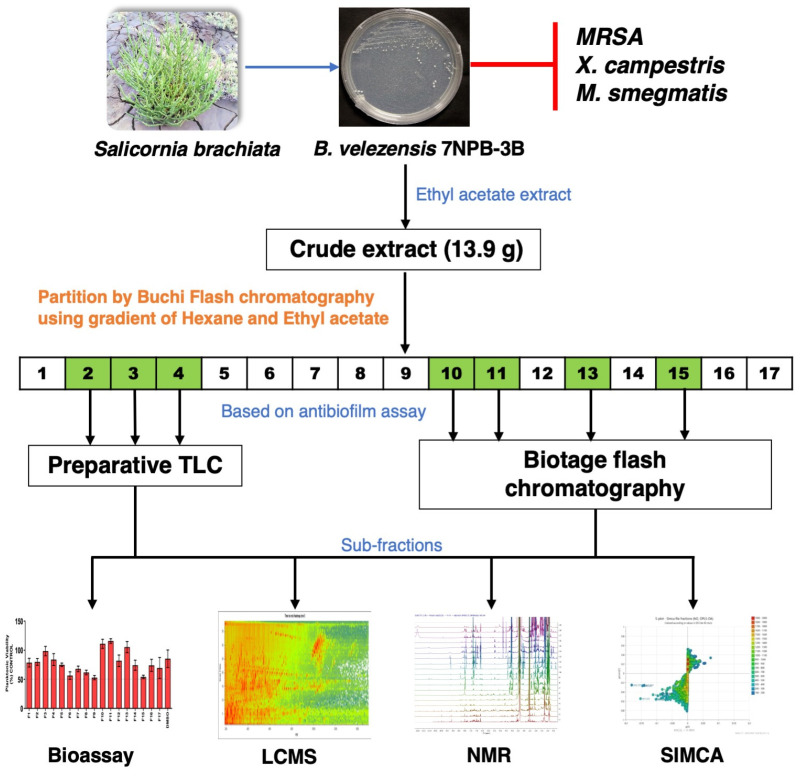
Isolation procedure of bioactive compounds from the endophytic bacteria *B. velezensis* 7NPB-3B.

**Figure 3:**
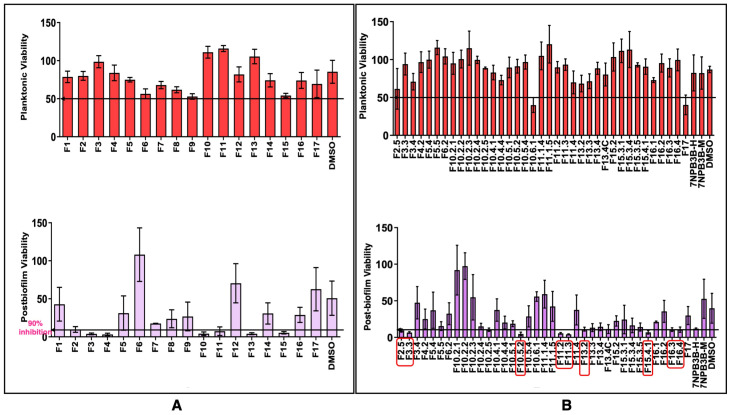
Bioassay against methicillin-resistant *S. aureus*. (**A**) Inhibition of planktonic MRSA (**top**) and biofilms (**bottom**) on treatment by fractions of *B. velezensis* 7NPB-3B. (**B**) Inhibition of planktonic MRSA (**top**) and biofilms (**bottom**) on treatment by the respective subfractions. The results shown were an average from three replicates. The red frames highlights the fractions with bioactivity > 90%.

The methanolic extract (13.4 g) was fractionated using normal phase flash chromatography resulting in 16 different fractions with the last fraction being the most polar (Scheme 1). To proceed with efficient isolation of bioactive metabolites, these fractions were tested for their inhibitory activity against planktonic cells of MRSA ATCC 43300 and for their capacity to destabilize the formed *MRSA* biofilms. Only two fractions F9 and F15 inhibited about 50% planktonic *MRSA* at a concentration of 100 µg/mL, whereas excellent dispersion of formed biofilms of up to 90% was observed with fractions F2, F3, F4, F10, F11, F13, and F15 (Scheme 1 and Figure 3A). Based on the results of the post-biofilm inhibition assay, the bioactive fractions were further purified by either preparatory TLC or Biotage flash chromatography. Moreover, the metabolite profile of the fractions was dereplicated using LC-HRMS before multivariate analysis. After the successive high-throughput chromatographic separation, a total of 37 subfractions were obtained. All the subfractions were tested for their anti-MRSA activity and post-biofilm inhibition of MRSA biofilms. Only two subfractions F10.6.1 and F17 exhibited activity >50% against planktonic MRSA cells at a concentration of 100 µg/mL. In the case of post-biofilm inhibition, nine subfractions, i.e., F2.5, F3.3, F10.5.2, F11.2, F11.3, F13.2, F15.4.1, F16.3, and F16.4, displayed >90% inhibition at the significant concentration of 100 µg/mL (Figure 3B). The pure fractions were then subjected to NMR analysis for identification and structure elucidation considering their bioactivity.

**Figure 4:**
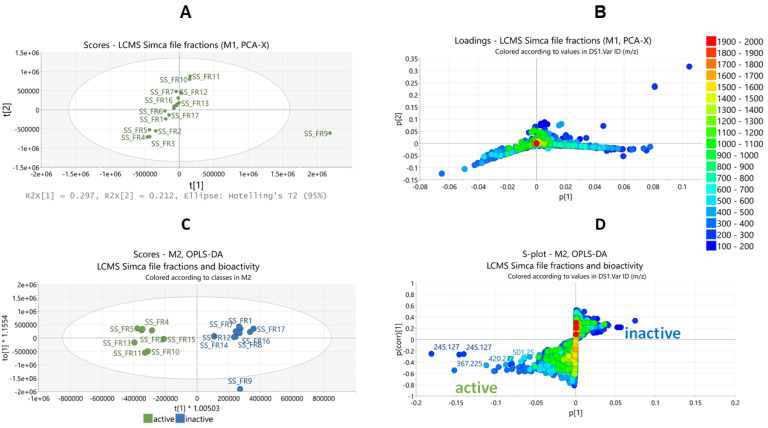
Multivariate analysis of the mass spectral data of fractions obtained from the organic extract of *Bacillus velezensis* 7NPB-3B. (**A**) Unsupervised principal component analysis (PCA) scores and (**B**) loadings plots show moderate separation between the datasets. (**C**) Supervised orthogonal projections to latent structures discriminant analysis (OPLS-DA) scores plot and (**D**) loadings S-plot differentiating the metabolites between active and inactive fractions listed in Table 2.

Secondary metabolites present in the *B. velezensis* 7NPB-3B were tentatively dereplicated using high-resolution LC-MS against the Dictionary of Natural Products (DNP version 2023) database. The relationship between the bioactive fractions and their metabolites was statistically evaluated using SIMCA. The unsupervised PCA scores plot showed the uniqueness and similarity of metabolites present in the fractions. Variable samples that clustered together indicated similarity in their chemical profile, while the dispersal of the fractions implied the differences. The farther the samples from the center axis of the scores plot, the higher the metabolite diversity for the respective fractions (Figure 4A). F9 was observed to be the outlier in the PCA analysis suggesting a unique chemical profile of the sample. The supervised multivariate analysis demonstrated by the OPLS-DA scores plots shown in Figure 4C depicted the differences between the two groups of fractions, F2, F3, F4, F5, F10, F11, F13, and F15 as the active fractions and F1, F6, F7, F8, F9, F12, F14, F16, and F17 for the inactive fractions. The active fractions were discriminated by ions peaks at *m*/*z* [M + H]^+^ 245.127 at 6.40 min, 296.148 at 6.77 min, 349.215 at 15.83 min, 367.225 at 17.10 min, 385.236 at 15.95 min, and 420.272 at 16.26 min, while for [M − H]^−^, discriminating ions peaks were observed at *m*/*z* 174.055 at 6.59 min, 447.239 at 16.23 min, and 501.250 at 14.78 min. Dereplication data of these ion peaks are listed in Table 2. It was noticeable that none of the surfactin congeners earlier detected from the TIC of the fractions were perceived as target metabolites for antibiofilm activity. Bioactive metabolites were detected between 200 and 500 Da with the occurrence of macrolactin congeners as the typical *Bacillus* secondary metabolites for ions peaks at *m*/*z* 385.236 [M + H]^+^ and 501.250 [M − H]^−^ [19].

**Figure 5:**
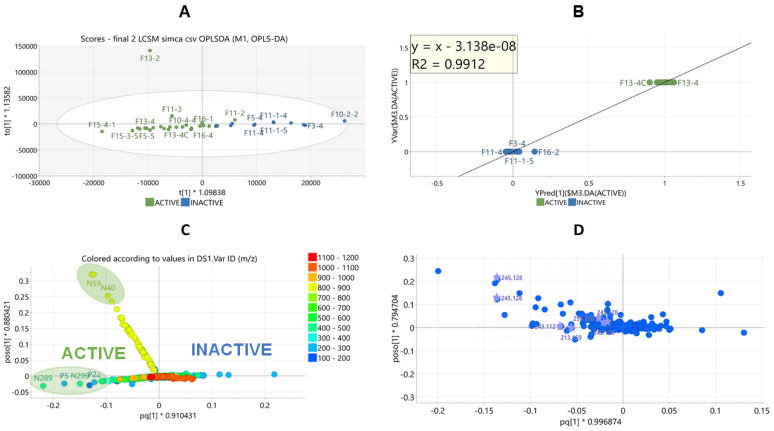
OPLS-DA of the mass spectral data of semi-purified subfractions of the active fractions shown in Figure 4. (**A**) Scores between active and inactive fractions. (**B**) The observed vs. predicted plot displays the observed versus predicted values of the active Y-variable. With a good model, all the points must fall close to this 45-degree line. The R2 of the regression line afforded a goodness of fit close to 1.0. (**C**) Loadings plot differentiating between the metabolites of active and inactive fractions. Encircled features represent the discriminating antibiofilm-active metabolites listed in Table 2. (**D**) Extracted loadings plot for metabolites between 200 and 300 Da to show the positions of the isolated compounds indicated by a star on the left quadrant for the active samples.

The antibiofilm compounds discriminated by multivariate analysis were dereplicated with DNP and listed in Table 2. The OPLS-DA scores plot of the subfractions indicated a unique chemistry for F13.2 (Figure 5A), which separated it from the rest of the active subfractions with a mass ion peak at *m*/*z* 863.06 [M − H]^−^; however, its molecular formula cannot be predicted following the seven heuristic rules [20]. In parallel to the separation of F13.2 from most of the active fractions, there was also an overlapping of weaker antibiofilm active fractions to the inactive right quadrant yielding low fitness R2 (0.65) and predictability Q2 (0.08) scores. The percentage variability scores were also low at only 0.04% between groups and 24% within groups, where the variability score must be higher between groups to indicate good separation. As presented in Table 3, the discriminating features from the active quadrant were not found in any of the fractions overlapping with those of the inactive quadrant; these subfractions (F2-5, F10-2-5, F10-4-4, F10-5-1, F10-5-2, F10-5-4, F11-2, F11-3, F13-3, F16-1, F16-4, and F17) were then excluded. This improved the R2 and Q2 scores to 0.99 and 0.46. Due to the low predictability score, this was validated through an observed vs. predicted plot (Figure 5B) to reveal the observed versus predicted values of the active Y-variable. Except for F11-4, F13-4, F13-4C, and F16-2, all the variables must fall close to the 45-degree line to attain a good model. But the R2 of the regression line afforded a goodness of fit of 0.99 cross-validating the predicted Y-variables for the active components.

The OPLS-DA loadings plot showed the distribution of discriminating metabolites at the ends of both active and inactive quadrants (Figure 5C), further confirming the bioactive masses to be present in the subfractions F15.4.1, F13.2, F10.2.4, F15.3.5, F3.3, F5.5, and F4.2. Amongst the detected antibiofilm features included diketopiperazines derivatives cyclo(tryptophanyltyrosyl), cyclo(isoleucylisoleucyl), and cyclo(isoleucylprolyl) or their corresponding leucyl isomers found at *m*/*z* 348.136 [M − H]^−^/350.149 [M + H]^+^, 227.174 [M + H]^+^, and 211.144 [M + H]^+^, respectively, compatible with the exact masses 349.143, 226.167, and 349.143 Da. *Bacillus* species have been reported to produce compounds of the diketopiperazines class [21,22,23]. The features present in the active quadrant also included the ion peak at *m*/*z* 447.239, putatively identified as Antibiotic L731128, earlier described from *Sporormiella intermedia* MG 5447 [24]. A related ion peak was preliminarily screened from the antibiofilm fractions FR3, FR4, and FR5 eluting at 16.23 min (Table 2). Another ion peak at *m*/*z* 557.331 [M + H]^+^ provided a compound hit for turnagainolide A, a known antibacterial earlier isolated from the marine-derived *Bacillus* sp. RJA2194 [25].

Meanwhile, the ion peaks at *m*/*z* 270.205, 469.258, and 471.273 remained unidentified after the dereplication of active fractions (Table 3 and Figure S1). The ion peak at *m*/*z* 270.205 [M + H]^+^ yielded a molecular formula prediction for C_11_H_23_N_7_O with a DBE of 4 for the exact mass of 269.197 Da. The ion peaks at *m*/*z* 469.258 [M − H]^−^ and 471.270 [M + H]^+^ eluting at 18.91 and 18.81 min, respectively, are compatible with the exact mass 470.265 with predicted molecular formulas of C_23_H_38_N_2_O_8_ (DBE = 6), C_24_H_34_N_6_O_4_ (DBE = 11), and C_28_H_38_O_6_ (DBE = 10) with an accuracy of 2.18, 1.80, and 3.91 ppm, respectively. A similar differentiating ion peak at *m*/*z* 469.258 [M − H]^−^ with a retention time of 18.84 min was detected from the TIC of the crude extracts (Table 1). The latter prediction of C_28_H_38_O_6_ (DBE = 10) was dereplicated as 7-*O*-(2*E*-butenoyl) macrolactin A, which was also previously reported from the marine-derived *Bacillus subtilis* B5 [19]. However, the proton NMR of the semi-purified fraction F15-4-1 did not match the structure of 7-*O*-(2*E*-butenoyl) macrolactin A.

The pre-defined bioactive metabolites obtained by multivariate analysis were able to guide the further purification and isolation of the antibiofilm compounds. During the purification step, the isolation of the compound with an ion peak at *m*/*z* 469.258 [M − H]^−^ and retention time at 18.8 min was targeted. In parallel, a series of diketopiperazine compounds and mixtures of other low-molecular-weight compounds ranging between 200 and 300 Da are being detected on the active fractions. These were again subjected to OPLS-DA, and these compounds were indeed positioning themselves on the active quadrants of the loadings plots as demonstrated in Figure 5D.

The study aimed to isolate antibiofilm compounds using metabolomics and bioassay-guided separation from the halophytic endophyte *B. velezensis* 7NPB-3B. Repeated chromatographic separation of the organic crude extract afforded a new metabolite (**1**) and four other previously isolated cyclic dipeptides (**2** to **5**). The structure elucidation and identification using 1D- and 2D-NMR with mass spectrometry led to identification of compounds **1** (*m*/*z* 469.358 [M − H]^−^)**,** cyclo-Phe-Leu (*m*/*z* 259.145 [M − H]^−^) **2**, cyclo-Phe-Val (*m*/*z* 245.128 [M − H]^−^) **3**, cyclo-Leu-Val (*m*/*z* 213.159 [M + H]^+^) **4**, and cyclo-Phe-Pro (*m*/*z* 243.112 [M − H]^−^) **5** (Figure 5). The presence of some cyclic dipeptides also known as diketopiperazines **2** to **5** was initially predicted through LC-MS dereplication. Thus, such results validated the accuracy of the dereplication process. The structures of the cyclic dipeptides were elucidated by 1D and 2D NMR as well as corroborated with the literature (Table S1 and Figure S2A) [26,27,28,29,30]. Comparison of the CD spectra of compounds **2** to **5** (Figure S2B) to those found in the literature followed the 2*S*,2′*S* (3S,6*S* for **2** to **4** and 3S,8a*S* for **5** in their IUPAC nomenclature) [31,32,33,34].

Compound **1**: Compound **1** (50 mg) was isolated as a dark brown oil, and its molecular formula was determined by high-resolution mass spectrometry as C_23_H_38_N_2_O_8_ based on the ion peak at *m*/*z* of 469.258 [M − H]^−^ with 6 degrees of unsaturation and an optical rotation of [α]^20^−23.60° (c 1.00, MeOH). The compound showed UV (MeOH) absorption maxima at 207 nm (log ε 4.24) and 260 nm (log ε 3.43).

The ^1^H NMR (DMSO*-d_6_*) analysis of compound **1** (Table 4) exhibited the presence of three spin systems. The ^1^H NMR spectrum (Figure S3) indicated the presence of a dipeptide group with characteristic alpha proton signal at δ_H_ 4.20 (m, H-2), two methylene group protons at δ_H_ 1.83 (m, H-4a), 2.04 (m, H-4b) and 2.89 (dtd, H-5a), 2.76 (m, H-5b), respectively, corresponding to a glutamic acid moiety terminating with a carboxylic acid group. Similarly, the other amino acid of the dipeptide corresponds to an unusual lysine unit detected with characteristic amine protons signals at δ_H_ 8.23 (d, *J =* 8.3 Hz), alpha proton at δ_H_ 4.18 (m, H-8), four methylene groups at δ_H_ 1.84 (m, H-10), 2.15 (ddt, H-11, H-12), 1.86 (m, H-11, H-12), and 3.38 (m, H-13). However, the amine group of lysine was instead substituted with an acid moiety. The downfield shift of the H-13 methylene group is due to the presence of the electron-withdrawing acid group at C-14. Both amino acids were connected to form a diketopiperazine ring (DKP). Furthermore, the broad singlet at δ_H_ 1.23 (H-17 to H-22) was observed indicating the presence of an alkyl fatty acid chain. Detailed analysis of DEPT NMR spectrum (Figure S4) along with HMBC spectrum (Figure S5) showed the presence of 5 carbonyl carbons at δ_C_ 165.2 ppm and 170.1 ppm corresponding to the amide bonds (C-1, C-7), in DKP ring as well for acid groups (C-6, C-14, C-24) along the side chains. Two alpha carbon signals of the two amino acids showed their characteristic carbon chemical shifts at 53.5 ppm (C-2) and 58.5 ppm (C-8). The methylene carbons of glutamic acid had δ_C_ resonances at 48.8 (C-4), 22.4 (C-5), and the same for a characteristic lysine unit with δ_C_ at 22.3 (C-10), 27.7 (C-11), 22.3 (C-12), and 44.9 (C-13). However, the downfield shifts of C-5, C-13, and C-23 pertained to the existence of a carboxylic acid in their periphery. The methylene groups for the fatty acid chain appeared at δ_C_ 28.9. ^1^H-^1^H COSY couplings (Figure S6) from H-2 to H-4 and H-5 along with HMBC correlations of H-4 to C-2, C-5, C-6 and H-2 to C-5 and C-1 confirmed the presence of glutamic acid. Similarly, COSY couplings from H-8 to H-9, H-10 to H-11, and H-12 to H-13 supported by HMBC correlations H-8 to C-7, C-10; H-10 to C-11; H-11 to C-7, C-8, C-12; H-12 to C-13, C-14; and H-13 to C-11, C-10 confirmed prevalence of the atypical lysine unit. The diketopiperazine ring formation is confirmed by the HMBC correlation of the H-9 amine proton of the unusual amino acid to C-1 and C-2, i.e., carbonyl and alpha carbon of glutamic acid, respectively. The four-bond HMBC correlation of H-16 and C-14 reveals the connection of the fatty acid alkyl chain with diketopiperazine moiety and cross-peaks from H-22 to C-24 further verify the carboxylic acid attached at the end of a saturated alkyl chain that is decanoic also known as capric acid. Capric acid (C10:0) has been reported to exhibit potent antibacterial activity against the Gram-negative bacteria *Chlamydia trachomatis* upon treatment with 10 mM of the compound [35].

**Figure 6:**
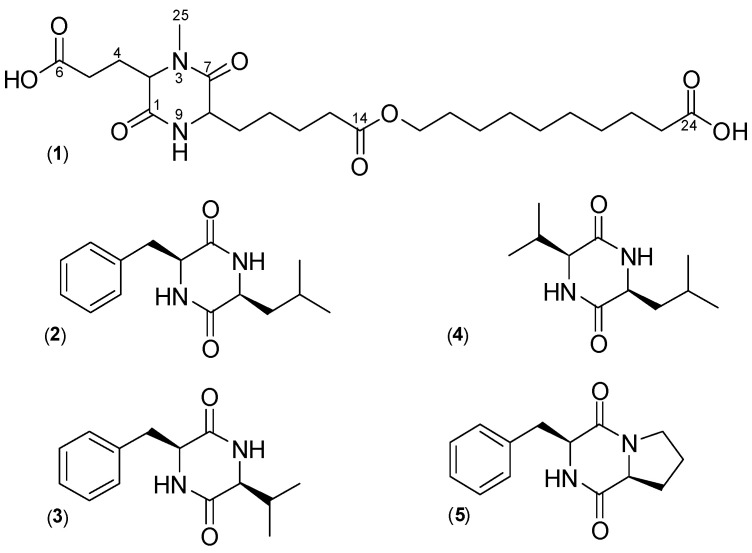
Structures of isolated compounds from *B. velezensis* 7NPB-3B.

**Figure 7:**
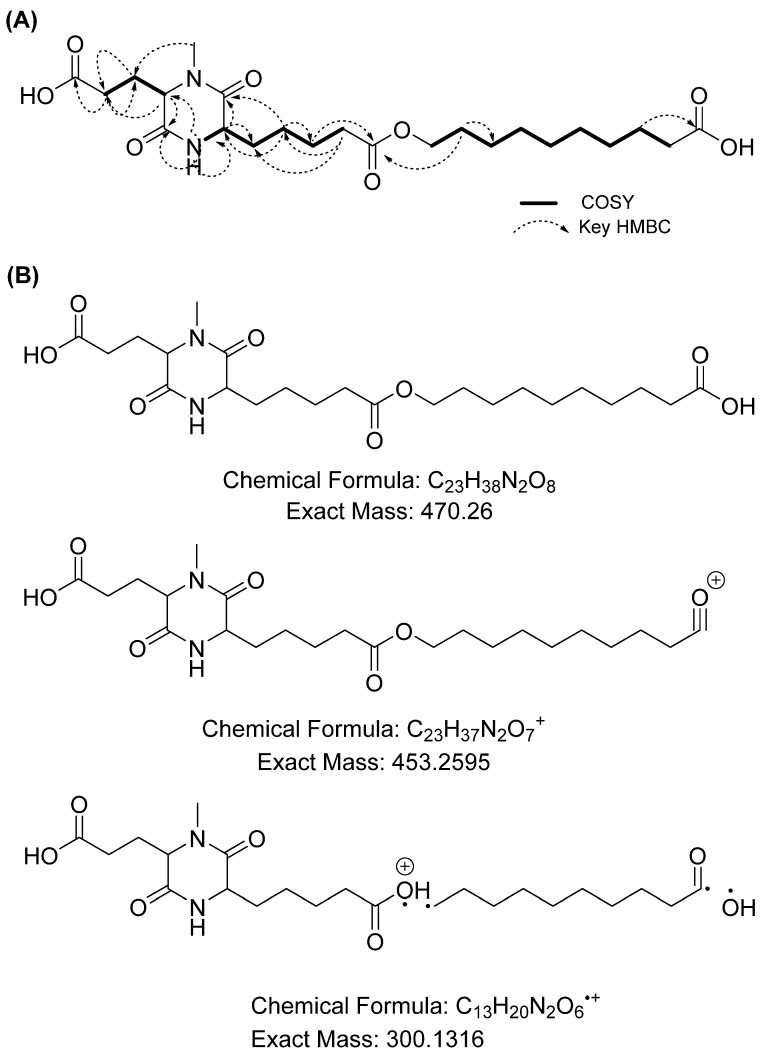
(**A**) COSY and key HMBC correlations of compound **1**. (**B**) The fragmentation pattern of compound **1** as obtained by MS/MS fragmentation confirming the structure of **1**.

The location of the methyl group was elucidated to be at N-3 based on the ^4^*J*-HMBC correlation from H-25 to C-4 (Figure 6, Figures S5 and S6). The chemical shifts of CH_2_-4 were typical to those of a γ-position to a carbonyl unit at δ_H_ 2.89 and 2.76, δ_C_ 48.8, which were downfield to that of the β-position that was shifted upfield at δ_H_ 2.04 and 1.83, δ_C_ 22.4. This was further corroborated by the presence of an amine proton on the atypical amino acid unit and its absence on the glutamic acid moiety [36,37]. The analysis of 1D- and 2D-NMR spectra confirmed the planar structure of **1** (Figure 6 and Figures S4–S8). The structure of **1** was further validated by the MS/MS fragmentation pattern that resulted in fragments *m*/*z* 453.26 and 299.25 (Figure 7B and Figure S8). Compound **1** was elucidated as 10-((5-(5-(2-carboxyethyl)-4-methyl-3,6-dioxopiperazin-2-yl)pentanoyl)oxy)decanoic acid. The ECD spectrum for compound **1** is shown in Figure S9.

**Figure 8:**
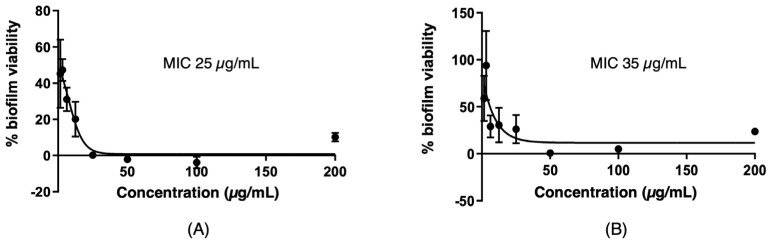
Post-biofilm MBEC determination of (**A**) **1** and (**B**) **5** against MRSA ATCC 43300.

Diketopiperazines are well-reported for their antibiofilm abilities. These molecules are reported to influence LuxR-mediated quorum sensing systems and offer an alternate mechanism for disrupting bacterial communication systems [38]. Compound **1** and cyclo-(Phe-Pro) **5** were found to exhibit biofilm dispersal ability at 100 µg/mL against MRSA biofilms via alamar blue assay. Upon further testing, the minimal biofilm eradication concentration (MBEC) for **1** and **5** was determined to be 25 µg/mL and 35 µg/mL, respectively, keeping the positive control ciprofloxacin with an MBEC of 3.125 mg/mL against MRSA ATCC 43300 (Figure 8 and Figure S10). The MBEC calculation validated that the **1** and **5** have a potent biofilm eradication capacity in comparison to the positive control and hence shall be tested further for the development as a lead molecule. Compounds **2** to **4** also exhibited post-biofilm inhibition potential, but the activities were not so noteworthy. Compound **5** contains proline amino acid in contrast to **2** to **4**; therefore, it can be noted that proline may be contributing towards the management of biofilms by diketopiperazines. There have been multiple reports where the diketopiperazine cyclo-(*L*-Phe*-L*-Pro) was found to modulate/interfere with the cell signaling of quorum sensing systems [39,40,41]. The ECD of compound **5** is comparable to that of a *L*, *L* configuration as reported by Domzalski et al. [31]. In another report, cyclo-(*L*-Phe*-L*-Pro) inhibited the Ptst promoter of *agr* quorum sensing system of *S. aureus* biofilms [42]. Furthermore, for the bioactivity against planktonic MRSA ATCC 43300 cells, subfraction F10.6.2 and fraction F17 demonstrated positive results. LC-MS of F10.6.2 indicated the presence of lipopeptide surfactins with masses (*m*/*z* 1006 [M − H]^−^, 1020 [M − H]^−^, 1034 [M − H]^−^, 1048 [M − H]^−^, 1074 [M + K]^+^, and 1088 [M + K]^+^), but they were not purified in this work. Surfactin analogs from *Bacillus* sp. are well known for their antibacterial potential [5]. Compounds like lipopeptide surfactins and an antimicrobial peptide *YS12* from *B. velezensis* exhibited antibiofilm activity against multiple clinical pathogens including MRSA [15] but were found inactive in our test organisms in this study. However, diketopiperazines with antibiofilm activities have not yet been reported from the endophytic *B. velezensis* strains. Furthermore, cyclic dipeptides reported from other strains of *B. velezensis* mostly exhibit bioactivity against fungal plant pathogens [43,44,45]. We report **1** (*m*/*z* 469 [M − H]^−^) and **2**–**4** for the first time from an endophytic *B. velezensis* strain. Therefore, the strain isolated from an untapped niche led to the identification of new compounds, confirming the effects of the environment on the chemical ecology of the bacteria.

## 3. Materials and Methods

### 3.1. Isolation and Identification of the Strain

The plant *Salicornia brachiata* Roxb. was collected from the salt marshland of New Port (21°45′15.7″ N, 072°14′01.4″ E) from Bhavnagar district, Gujarat, India. The isolation and identification of endophytes are described in the work by Singh et al. (2021). Briefly, the plant material was surface-sterilized, cut aseptically, and then inoculated in 8 different media for the growth of diverse bacteria. The isolates were screened based on their bioactive potential against a panel of pathogens. Selected bioactive endophytes were further identified using 16s rRNA sequencing [5].

### 3.2. Fermentation and Extraction

The large-scale liquid-state fermentation of *B. velezensis* 7NPB-3B was carried out to obtain the crude extract for the isolation of compounds. The seed broth of the strain was prepared using Luria Bertani (LB) broth media (1.0% Tryptone, 0.5% yeast extract, 1.0% sodium chloride), pH (7.0–7.5), in a 500 mL conical flask and incubated at 30 °C, 150 rpm for 24 h. Thereafter, a 5% (*v*/*v*) concentration of seed culture was used to inoculate 60 × 1 L conical flasks containing 400 mL of LB production medium with final pH 7 (autoclaved at 121 °C, 15 min). The culture was incubated at 30 °C, 150 rpm for 5 days wherein the incubation period was decided based on time-dependent antimicrobial activity against *S. aureus* MCC 2043. Thereafter each flask was extracted with an equal amount of ethyl acetate to partition the organic metabolites of culture into the solvent. The organic layer obtained was pooled and concentrated using a rotary evaporator to obtain the crude extract.

### 3.3. Isolation of the Bioactive Metabolites

The crude extract (13.9 g) was partitioned with a solution of 10% n-hexane and 90% methanol to remove fatty acids. The extract containing methanol-soluble compounds (13.40 g) was collected for further isolation work. The fractionation of the methanol extract was accomplished using MPLC (Buchi Flash, Flawil, Switzerland). Linear gradient elution was employed with ethyl acetate (A), hexane (B), and methanol (C) as the mobile phase at a flow rate of 100 mL/min. The sample was mixed with celite and loaded on top of a pre-packed silica column (20–45 μm, 23 × 110 mm, Silica VersaPak cartridge, Sigma Aldrich, St. Louis, MO, United States [2]). It was connected to a Buchi Pump Manager C-615 coupled to binary pumps (Buchi Modules C-601, Flawil, Switzerland); 100% B was run for 5 min, followed by 100% B to 100% A for 60 min, and finished with 100% B for the last 15 min. Still, the extract color was visible in celite; therefore, an extra wash of 15 min was given with a mobile phase of 90% A and 10% C. The total run time was 95 min. Fractions were collected manually for every 30 s, i.e., 50 mL in collecting flasks. Fractions were run on silica TLC plates (TLC Silica gel 60 RP-18 F254s, Merck, Darmstadt, Germany), visualized in UV light (λ = 254 nm), and the ones with similar TLC profiles were pooled together, yielding a total of 16 fractions. The fractions were analyzed using LC-MS for dereplication study and tested for biofilm inhibition. Depending on the weights obtained and the bioactivity of the fractions, further purification was performed. Fractions 2 to 5 were further separated using preparative TLC, and other bioactive fractions were purified with Biotage high-throughput flash chromatography (Biotage Isolera One, Uppsala, Sweden) using normal phase pre-packed column (SNAP, Biotage Sweden Ab, Biotage, LLC, MORE, Uppsala, Sweden). Finally, a total of 36 subfractions were obtained after consecutive fractionation, which were analyzed for their biofilm inhibition properties. The structure elucidation was performed for the purified compounds using 1D NMR and 2D NMR (Bruker ^1^H 400 MHz, ^13^C 150 MHz) spectroscopy. Samples were prepared by dissolving 5 mg of bacterial extract or fractions in 600 μL DMSO-d6 (Sigma-Aldrich, Dorset, UK). Spectra were processed using MestReNova (Mnova 14.2.0) software (Mestrelab Research, Santiago de Compostela, Spain).

Data-dependent MS^2^ and MS^3^ experiments were carried out using a Finnigan LTQ Orbitrap coupled to a Surveyor Plus HPLC pump (Thermo Scientific, Bremen, Germany) and autosampler (Thermo Fisher, Bremen, Germany) in positive and negative ionization modes using a mass range of *m*/*z* 100–2000 and 30,000 resolutions. The capillary temperature was 270 °C, the ion spray voltage was 4.5 kV, the capillary voltage was 35 V, the tube lens voltage was 110 V, and the sheath and auxiliary gas flow rates were 50 and 15, respectively (units not specified by the manufacturer). Multi-fragmentation (MS*^n^*) experiments were accomplished on an Orbitrap analyzer. CID (collision-induced dissociation) was utilized with a normalized collision energy of 35%, activation Q of 0.250 ms, and activation time of 30,000 ms applied on ions of most intense, second most intense, and third most intense peaks for MS^2^ and MS^3^, respectively, at an isolation width of 3 microns with 5 microscans. Resolution was at 15,000 m/Δm50%, while the minimum ion signal threshold was set to 500. Fragment mass tolerance for molecular formula detection was set at ±5 ppm.

### 3.4. In Vitro Biofilm Inhibition Assay

AlamarBlue^®^ biofilm viability assay was adopted to check the antibiofilm potential of fractions of *B. velezensis* 7NPB-3B obtained after MPLC separation. Stock solutions of each of the extract/fraction were prepared with 1 mg of the samples dissolved in 100 μL of biological-grade DMSO to get a concentration of 10 μg/μL. Assay plates were prepared by adding 200 μL of LB broth pre-inoculated with log phase culture (1 × 10^6^ cfu/mL) of biofilm-forming *Staphylococcus aureus* MRSA ATCC 43300. A control containing 200 μL of LB without inoculation by the pathogen was maintained. The plates were incubated at 35 °C in a shake incubator at a speed of 140 rpm for 24 h. After 24 h, the planktonic cells were removed carefully by pipetting and washed with sterile 0.1 M PBS such that the formed biofilms were not distorted. Thereafter, the plate was seeded with subfractions such that the final concentration of the sample in each well was 100 μg/mL and the volume was made up to 200 μL by adding LB media. DMSO was used as a negative control, while ciprofloxacin served as a positive control. Finally, the newly seeded plate was placed into a shaking incubator and agitated for 23 h, at 35 °C, 200 rpm. After incubation, the wells were rinsed with 0.1 M PBS such that the formed biofilms were not distorted. After washing, 90 μL of DMEM and 10 μL of AlamarBlue^®^ were added to the wells. The plates were further incubated for 1.5 h in a shaking incubator at 35 °C covered with foil. Absorbance reading was taken at 560 nm excitation wavelength and 590 nm emission wavelength after incubation. The readings were processed in an EXCEL sheet, and final graphs were produced after processing the results with GraphPad Prism 10.1.0.

Similarly, compounds **1** and **5** were further assayed to determine their MBEC values against biofilm-forming MRSA (ATCC 43300). A dilution plate was prepared for each compound with a concentration range of 200 to 1.56 µg/mL by double dilution method. Ciprofloxacin was used as a positive control, with a concentration range of 100 to 0.78 µg/mL. The MBEC values were determined using graph pad prism 10.1.0.

### 3.5. LC-MS-Dependent Metabolomics and Dereplication

#### 3.5.1. LC-HRMS

For LC-MS analysis, a 1 mg/mL sample was prepared in a 1:1 MeOH–ACN solvent system. Experiments were carried out using an Exactive mass spectrometer with an electrospray ionization source attached to an Accela 600 HPLC pump with an Accela autosampler and UV/Vis detector (Thermo Scientific, Bremen, Germany). The mass accuracy was set to less than 3.0 ppm. The Orbitrap mass analyzer can limit the mass error to ±3.0 ppm. Mass spectrometry was carried out over a mass range of 100–2000 *m*/*z* in positive and negative ionization modes with a spray voltage of 4.5 kV and capillary temperature of 270 °C. About 10 μL was injected from each vial, at a flow rate of 300 μL/min. The column used was an ACE5 C18 column (5 μm × 75 mm × 3 mm) (Hichrom Limited, Reading, UK). A binary gradient method was utilized using the two solvents, A (water and 0.1% formic acid) and B (MeCN and 0.1% formic acid). The gradient was carried out for 45 min and the program followed; at zero minutes A = 90% and B = 10%, at 30 min A = 0% and B = 100%, at 36 min A = 90% and B = 10% until end at 45 min. The UV absorption wavelength was set at 254 nm, the sample tray temperature was maintained at 4 °C, and the column at 20 °C. The samples were run sequentially, with solvent and media blanks analyzed first. LC-MS data were acquired using Xcalibur version 2.2.

#### 3.5.2. Metabolic Profiling Studies

LC–MS data were used for metabolomic profiling studies following the protocol of Macintyre et al. (2014) [46]. Raw data were first converted to mzML file format using the software ProteoWizard 3 [47]. Data processing was carried out using the software MZMine 3 [48,49]. The data were processed while maintaining the specific parameters as mentioned (mass detector: centroid; noise level: 10,000; minimum time span: 0.2 min; minimum height: 1 × 106; *m*/*z* tolerance: 0.001 *m*/*z* or 5 ppm), deconvolution (chromatographic threshold: 5%), deisotoping (*m*/*z* tolerance: 0.001 *m*/*z* or 5 ppm, retention time (RT) tolerance: 0.1 absolute (min), maximum charge: 2, representative isotope: most intense), filtering (*m*/*z* range: 100–1999, RT: 0–45 min), alignment (*m*/*z* tolerance: 0.001 *m*/*z* or 5 ppm, *m*/*z* weight: 20, RT tolerance: 5 relative %), and gap filling (*m*/*z* tolerance: 0.001 *m*/*z* or 5 ppm, intensity tolerance: 1%). Complete software settings were as described by Macintyre et al. [46]. The positive- and negative-ionization data were processed together in an Excel macro that minimized the risk of missing poorly ionized compounds. The Excel macro was used to dereplicate the samples, matching each *m*/*z* found in the samples with those in the Dictionary of Natural Products (DNP, version 2017) database to provide complete information of all the putatively identified metabolites, as well as those that were unidentified. For the dereplication, a feature ID number, ionization mode, *m*/*z*, retention time, possible molecular formula, peak intensity, related compounds, and source of the specific metabolites (if available) were generated.

#### 3.5.3. Multivariate Analysis

Multivariate analyses (MVAs) that included both principal component analysis (PCA) and orthogonal projection to latent structures discriminant analysis (OPLS-DA) for the prediction of antibacterial metabolites were carried out using the software SIMCA ver. 17 (Umetrics, Umeå, Sweden). PCA was used to observe an overview of the variance of secondary metabolites between different fractions generated from LC–MS data and to detect outliers that could influence the model. OPLS-DA was also used to identify chemically distinct samples that may yield novel and bioactive secondary metabolites. A pareto scaling was applied to all MVAs to reduce the influence of intense peaks while emphasizing weaker peaks that may have more biological relevance [50]. The Q2 and R2 values were reported as a qualitative measure of consistency between the predicted and original data. These values explained the goodness of the prediction of the statistical models, representing the total explained variance and the predictive power of the models.

## 4. Conclusions

The study presents the isolation of antibiofilm compounds from endophytic *B. velezensis* 7NPB-3B wherein the targeted isolation of compounds was aided by LC-MS-based metabolomics, dereplication using DNP database, and bioactivity-guided fractionation. To the best of our knowledge, this is the first study on the application of metabolomics and dereplication for the isolation of bioactive compounds from endophytes of *S. brachiata*. The targeted bioactive fractions were also backed by multivariate analysis, such as PLS-DA and OPLS-DA. Compounds of class diketopiperazine were isolated from the source that exhibited prominent inhibition of biofilms formed by an MRSA pathogen. A novel diketopiperazine derivative (**1**) along with four known compounds was isolated from the endophyte. These are the first reports of these compounds from the endophytic *B. velezensis*. The work justifies the investigation of new drug lead molecules from known sources as well as untapped niches with the help of the modern omics approach.

## Figures and Tables

**Table 1:** The predicted compounds from the TIC of the crude extract of *B. velezensis* 7NPB-3B after dereplication using the DNP database. The listed features are arranged according to their retention time (RT) in min, while the selected masses are the peaks visible in the TIC. Highlighted features in grey were hits only found in plants, which may instead indicate an unknown compound. The highlighted row in green indicates the isolated metabolite in this study.

Mzmine ID *	RT (min)	MS *m*/*z*	Exact Mass	Chemical Formula	Name	Tolerance	Source	Peak Area
P48233	1.19	170.084	169.076	C_7_H_12_N_3_P	No hits			
P21878	1.50	212.091	211.084	C_10_H_13_NO_4_	*N*-salicyloylserinol	1.12	*Streptomyces hygroscopicus*	2,076,169.45
P20118	4.65	211.148	210.140	C_16_H_18_	1,7-hexadecadiene-10,12,14-triyne	0.006	Flower heads of *Chrysanthemum leucanthemum*	2,859,745.14
P25741	6.66	213.148	212.141	C_12_H_20_O_3_	10-hydroxy-10-methyl-2-undecen-4-olide	0.55	Marine bacteria *Streptomyces* strain MO2750, *Streptoverticillium luteoverticillatum* strain 11014	738,381.18
N12863	6.93	284.130	285.138	No hits	No hits			376,501.26
P48148	7.65	247.148	246.140	C_19_H_18_	1,7-diphenyl-1,3,5-heptatriene	−0.10	Rhizomes of *Curcuma comosa*	120,097.45
P46440	8.24	227.179	226.172	C_17_H_22_	1,3,7,9,13-heptadecapentaen-11-yne	−0.52	Various Asteraceae species	75,738.67
N22740	8.76	261.149	262.157	C_16_H_22_O_3_	Paecilocin A or D Spartinoxide Solanapyrone H Versiol	1.02	Marine-derived *Paecilomyces varioti* Marine-derived *Phaeosphaeria spartina* Marine-derived *Microsphaeropsis* sp. Stamm 6288 Marine-derived *Aspergillus versicolor*, *Penicillium* decumbens, *P.* striatisporum	537,461.67
P1561	10.79	250.112	249.104	C_10_H_21_NO_2_P_2_ C_3_H_11_N_11_O_3_	No hits			2.16 × 10^8^
P259	14.24	367.226	366.219	C_24_H_30_O_3_	Diorcinol K	−0.79	Marine-derived *Aspergillus* sp. CUGB-F046	2.25 × 10^9^
N23146	15.07	501.251	502.258	C_30_H_30_N_8_	No hits			1.61 × 10^9^
N2762	16.88	559.283	560.290	C_31_H_45_O_7_P	Oxydifficidin Proticin	0.03	*Bacillus subtilis*	4.99 × 10^8^
N53	18.84	469.258	470.265 3.91 ppm 1.80 ppm 2.18 ppm	C_28_H_38_O_6_ DBE = 10 C_24_H_34_N_6_O_4_ DBE = 11 C_23_H_38_N_2_O_8_ DBE = 6	7-*O*-(2*E*-butenoyl) macrolactin A No hits No hits	−0.19	Marine-derived *Bacillus subtilis* B5	5.39 × 10^9^
P10773	20.10	154.990	153.982	C_6_H_3_O_3_P	No hits			274,191.89
P34624	20.77	358.331	357.324	C_21_H_43_NO_3_	2-amino-heneicosene-1,3,4-triol	−0.58	*Helichrysum cameroonense* *Parinari hypochrysea* *Acnistus arborescens*	14,120.53
P14139	23.81	447.325	446.318	C_31_H_42_O_2_	8′-apo-β-caroten-8′-al; 8′-carboxylic acid, Me ester	−0.27	*Staphylococcus aureus*	8.37 × 10^8^
P41851	25.36	447.325	446.318	C_31_H_42_O_2_	Same as P14139	−0.10	*Staphylococcus aureus*	5.59 × 10^8^
P38446	26.33	994.643	993.636	C_50_H_87_N_7_O_13_	Surfactin B1, 4-L-alanine analog	0.50	*Bacillus subtilis* S499	3.31 × 10^8^
P1275	27.32	1008.659	1007.652	C_51_H_89_N_7_O_13_	Surfactin C1, 4-L-alanine analog; Surfactin A1,A2,A3	0.70	*Bacillus subtilis* S499	1.67 × 10^9^
P10881	27.7	1008.659	1007.652	C_51_H_89_N_7_O_13_	Surfactin C1, 4-L-alanine analog; surfactin A1,A2,A4	0.59	*Bacillus subtilis* S500	4.70 × 10^8^
P1149	28.6	256.263	255.256	C_16_H_33_NO	11*E*,12-dihydro-2*S*-amino-11,15-hexadecadien-3*R*-ol	−0.70	*Pseudodistoma obscurum*	3.61 × 10^7^
P211	28.89	1022.675	1021.667	C_52_H_91_N_7_O_13_	Surfactin C1, 7-L-valine analog, surfactin B1, B2	0.49	*Bacillus subtilis* and *Bacillus pumilus* KMM 456	2.16 × 10^9^
P310	29.27	1022.675	1021.667	C_54_H_98_N_5_O_7_P_3_ C_44_H_97_N_9_O_13_P_2_ C_49_H_100_NO_18_P C_47_H_88_N_15_O_8_P C_49_H_95_N_13_O_2_P_4_ C_64_H_99_NOP_4_ C_56_H_105_N_3_OP_6_ C_57_H_89_N_11_O_2_P_2_ C_52_H_91_N_7_O_13_ C_46_H_104_N_7_O_7_P_5_	No hits			3.51 × 10^9^
P6562	30.13	1034.675	1033.668	C_57_H_105_N_3_OP_6_ C_50_H_95_N_13_O_2_P_4_ C_32_H_13_N_2_O_9_P_15_ C_47_H_104_N_7_O_7_P_5_ C_45_H_97_N_9_O_13_P_2_ C_28_H_21_N_4_O_2_P_19_ C_55_H_98_N_5_O_7_P_3_ C_54_H_87_N_11_O_9_ C_42_H_101_N_15_O_2_P_6_ C_50_H_100_NO_18_P	No hits			2,879,678.11
P6206	31.14	1036.691	1035.684	C_53_H_93_N_7_O_13_	Surfactin B2, 1-Me ester, surfactin C1 7-L-isoleucine analog, C2 7-L-valine analog	0.88	*Bacillus* sp.	1.12 × 10^8^
P15049	32.6	415.357	414.349	C_28_H_46_O_2_	7-methoxycholesta-5,22-dien-3-ol	0.41	Bryozoa *Cryptosula pallasiana*	318,610.49
P24946	37.61	217.158	216.151	C_15_H_20_O	Anaephene C	−1.40	Marine-derived *Hormoscilla* sp.	1,503,069.04

* The letters P and N represent the ionization mode.

**Table 2:** Dereplicated antibiofilm target metabolites from the OPLS-DA loadings plot of bioactive fractions using DNP. The highlighted row indicates the isolated and elucidated compound in this study. * The letters P and N represent the ionization mode.

Primary ID *	*m*/*z*	RT (min)	Fraction Found	Exact Mass	Molecular Formula	Dereplicated Compound	Biological Source
N2994	174.055	6.59	FR4	175.0622	No prediction		
P6029	245.127	6.40	FR10 FR11	244.12051	C_14_H_16_N_2_O_2_	Cyclo(phenylalanylprolyl) (**5**)	Produced by the marine bacterial strains CF-20 and C-148 obtained from molluscs
P29373	296.148	6.77	FR4	295.1411	C_15_H_21_NO_5_	Coronafacic acid; L-serine amide	Produced by *Pseudomonas syringae*
P55	349.215	15.83	FR3 FR4 FR5	348.2075	C_18_H_33_ClO_4_	10*R*-chloro-plakortether B	*Plakortis simplex*
P16	367.225	15.93	FR3 FR4 FR5	366.2181	C_24_H_30_O_3_	Diorcinol K	Marine-derived *Aspergillus* sp. CUGB-F046
P239	385.236	15.37	FR3	384.2286	C_24_H_32_O_4_	7,13-epoxymacrolactin A	Marine-derived *Bacillus subtilis B5*
P6477	420.272	16.26	FR3	419.2652	C_24_H_37_NO_5_	Aspochalasin E	Marine-derived *Aspergillus* sp. *XS-2009-0B15*
N3227	447.239	16.23	FR3 FR4 FR5	448.2467	C_25_H_36_O_7_	Antibiotic L 731128	*Sporormiella intermedia* MG 5447
N26607	501.25	14.78	FR5	502.2576	C_28_H_38_O_8_	7-succinoyl-macrolactin A	*Bacillus amyloliquefaciens NJN-6*, *B. subtilis DSM 16696*, *marine Bacillus* sp. *Sc026*

**Table 3:** Dereplicated antibiofilm target metabolites from the OPLS-DA loadings plot of subfractions using DNP. Features are arranged and listed according to their ion peaks at *m*/*z* in Da. Highlighted rows indicate the isolated and elucidated compounds in this study. Structures of dereplicated compounds are shown in Figure S1.

Primary ID *	*m*/*z*	RT (min)	Subfraction	Exact Mass	Molecular Formula	Dereplicated Compound	Biological Source
P5	211.144	5.14	F6-2	210.136	C_11_H_18_N_2_O_2_	Cyclo(isoleucylprolyl)	Marine-derived *Pseudomonas aeruginosa* and *Vibrio parahaemolyticus*
							Marine bacterial strains CF-20 and C-148 isolated from larvae of the mollusc *Pecten maximus*
P22	211.144	5.19	F6s	210.136	C_11_H_18_N_2_O_2_	Same as P5	
P151	227.174	7.41	F4-2	226.167	C_12_H_22_N_2_O_2_	Cyclo(isoleucylisoleucyl)	Marine-derived *Paecilomyces marquandii*
P185	227.175	8.12	F10-2-4	226.167	C_12_H_22_N_2_O_2_	Same as P151	
P318	270.205	10.76	F5-5	269.197	C_11_H_23_N_7_O DBE = 4	No hits	
N32	348.136	6.75	F15-3-5	349.143	C_20_H_19_N_3_O_3_	Cyclo(tryptophanyl-tyrosyl)	Marine-derived *Aspergillus niger* EN-13 and from *B. subtilis* B38
P108	350.149	6.75	F15-3-5	349.142	[M + H]^+^ of N32	Same as N32	
N157	447.239	14.21	F5-5	448.246	C_25_H_36_O_7_	Antibiotic L 731128	*Sporormiella intermedia* MG 5447
N289	469.258	18.54	F15-4-1	470.265	Same as N299		
N299	469.258	18.91	F15-4-1	470.265	[M − H]^−^ of P802	No hits	
P802	471.273	18.81	F15-4-1	470.265 (Δppm) (2.18) (1.80) (3.91)	C_23_H_38_N_2_O_8_ DBE = 6 C_24_H_34_N_6_O_4_ DBE = 11 C_28_H_38_O_6_ DBE = 10	No hits No hits 7-*O*-(2*E*-butenoyl) macrolactin A	Marine-derived *Bacillus subtilis* B5
P611	557.331	15.98	F3-3	556.324	C_30_H_44_N_4_O_6_ DBE = 11	Turnagainolide A	Marine-derived *Bacillus* sp. RJA2194 and *Microascus* sp. 098059A. *Arthrobacter* sp. PGVB1 and *Streptomyces rutgersensis* T00
N40	863.067	7.12	F13-2	864.074	No prediction		
N59	863.067	8.26	F13-2	864.074	No prediction		

* The letters P and N represent the ionization mode.

**Table 4:** ^1^H NMR (400 MHz) and ^13^C NMR (150 MHz) data of compound 1 (DMSO-*d*_6_).

SN	Type	δ_H_ (ppm, Mult. J HZ)	δ_C_ (ppm)	HMBC
1	CO		165.2	
2	CH	4.20 (*m*)	53.5	C1, C4, C5
3	N			
4	CH_2_	2.89 (dtd, *J*= 12.9, 10.6, 10.7, 5.6 Hz) 2.76 (*m*)	48.8	C-2, C-5, C-25
5	CH_2_	2.04 (*m*) 1.83 (*m*)	22.4	C-2, C-4, C-6
6	CO		165.2	
7	CO		170.1	
8	CH	4.18 (*m*)	58.5	C-9, C-11
9	N*H*	8.23 (*d*, *J* = 8.3 Hz)		C-1, C-2, C-8, C-10
10	CH_2_	1.84 (*m*)	22.3	C-8, C-9, C-11
11	CH_2_	2.15 (ddt, *J* = 11.5, 9.8, 4.0 Hz) 1.86 (*m*)	27.7	C-7, C-8, C-10, C-12
12	CH_2_	2.15 (ddt, *J* = 11.5, 9.8, 4.0 Hz) 1.84 (*m*)	22.3	C-13, C-14
13	CH_2_	3.38 (*m*)	44.9	C10, C-11, C-12
14	CO		170.1	
15	CH_2_	3.31 (*m*)	62.3	
16	CH_2_	1.83 (*m*)	22.4	C-14, C-17
17	CH_2_	1.23 (*s*)	28.9	
18	CH_2_	1.23 (*s*)	28.9	
19	CH_2_	1.23 (*s*)	28.9	
20	CH_2_	1.23 (*s*)	28.9	
21	CH_2_	1.23 (*s*)	28.9	
22	CH_2_	1.23 (*s*)	28.9	C-24
23	CH_2_	3.38 (*m*)	44.9	
24	CO		170.1	
25	CH_3_	2.54 (*s*)	37.9	C-4

## Data Availability

No new data were created or analyzed in this study. Data sharing is not applicable to this article.

## Supplementary Materials

The following supporting information can be downloaded at https://www.mdpi.com/article/10.3390/microorganisms12020413/s1, Figure S1: Structures of the discriminating dereplicated metabolites detected from the OPLS-DA plot of subfractions listed in the Table 3; Figure S2A: 2D correlations (COSY and HMBC) of compounds **2**–**5**; Figure S2B: ECD spectra of compounds **2** to **5** in methanol; Figure S3: ^1^H NMR spectrum of **1**; Figure S4: J Mod ^13^C NMR spectrum of **1**; Figure S5: HMBC spectrum of **1**; Figure S6: COSY spectrum of compound **1** in methanol; Figure S7: HSQC spectrum of **1**; Figure S8: LC-HRMS and MS/MS fragmentation of **1**; Figure S9: ECD spectrum of compound **1**; Figure S10: Post-biofilm MBEC determination of ciprofloxacin against MRSA ATCC 43300; Table S1: ^1^H- and ^13^C- NMR data of isolated compounds **2**–**5** (DMSO-*d_6_*, 400 Hz).
