# Supplementary material for: Targeted Isolation of Antibiofilm Compounds from Halophytic Endophyte Bacillus velezensis 7NPB-3B Using LC-HR-MS-Based Metabolomics
Source: Microorganisms. 2024 Feb 19;12(2):413. doi: 10.3390/microorganisms12020413 (PMC10891937; doi:10.3390/microorganisms12020413)

Targeted Isolation of Antibiofilm Compounds from Halophytic Endophyte *Bacillus velezensis* 7NPB-3B using LC-HR-MS based Metabolomics

Sanju Singh[1,2,3], Elizabeth Nwagwu[3], Louise Young[3], Pankaj Kumar[1,2], Pramod B. Shinde[1,2,*], RuAngelie Edrada-Ebel[3,*]

[1]Natural Products & Green Chemistry Division, CSIR-Central Salt and Marine Chemicals Research Institute (CSIR-CSMCRI), Council of Scientific and Industrial Research (CSIR), Bhavnagar-364002, Gujarat, India

[2]Academy of Scientific and Innovative Research (AcSIR), Ghaziabad-201002, India

[3] Strathclyde Institute of Pharmacy and Biomedical Sciences, University of Strathclyde, The John Arbuthnott Building, 161 Cathedral Street, Glasgow G4 0RE, UK

* Correspondence: pramodshinde@csmcri.res.in (P.B.S.); ruangelie.edrada-ebel@strath.ac.uk (R.E.E)

Table S1. ^{1}H- and ^{13}C-NMR data of isolated compounds **2-5** (DMSO-*d_6*, 400Hz)

Sr. no.	**2**		**3**		**4**		**3**	
	^{1}H NMR δ_H **(ppm, multi. *J* in Hz)**	^{13}C NMR, $\delta_{H\ (ppm)}$	^{1}H NMR δ_H **(ppm, multi. *J* in Hz)**	^{13}C NMR	^{1}H NMR δ_H **(ppm, multi. *J* in Hz)**	^{13}C NMR	^{1}H NMR δ_H **(ppm, multi. *J* in Hz)**	^{13}C NMR
	**Leucine**		**Valine**		**Leucine**		**Proline**	
1		167.88 (C)	-	165.53 (C)	-	168.61 (C)	-	169.00 (C)
2	3.66 (*dt*, *J* = 9.5, 4.8 Hz, 1H)	55.75 (CH)	3.62 (*ddd*, *J* = 4.0, 2.7, 1.1 Hz, 1H)	59.96 (CH)	3.77 (*m*, 1H)	52.80 (CH)	4.07 (*dd*, *J* = 9.9, 7.0 Hz, 1H)	58.12 (CH)
3	δ 8.32 (*dd*, *J* = 7.5, 4.2 Hz, 1H)	-(NH)	8.04 (*d*, *J* = 2.5 Hz, 1H)	- (NH)	8.17 (*d*, *J*= 2.67, 1H)	- (NH)		- (NH)
4	1.54 (*td*, *J* = 8.0, 3.1 Hz, 1H) 1.81 (*m*, 1H)	45.21 (CH_2)	2.15 – 2.05 (m, 1H)	31.97 (CH)	1.63 (*m*, 1H) 1.45 (ddd, *J*= 13.80, 8.60, 5.46, 1H)	44.55 (CH_2)	3.26 (*dd*, *J* = 11.8, 6.2 Hz, 2H)	44.36 (CH_2)
5	1.81 (*m*, 1H)	24.37 (CH)	0.95 (*d*, *J* = 7.0 Hz, 3H)	19.14 (CH_3)	1.84 (*q*, *J*= 7.34, 1H)	24.04	1.72 (*td*, *J* = 8.3, 4.2 Hz, 2H)	21.56 (CH_2)
6	0.87 (*d*, *J* = 6.2 Hz, 3H)	22.09 (CH_3)	0.86 (*d*, *J* = 4.6 Hz, 3H)	17.79 (CH_3)	0.89 (*d*, *J*= 6.62, 3H)	23.48 (CH_3)	2.01 (*dq*, *J* = 11.7, 5.4 Hz, 1H) 1.43 (*td*, *J* = 12.9, 6.6 Hz, 1H)	27.55 (CH_2)
7	0.90 (*d*, *J* = 6.6 Hz, 3H)	22.43 (CH_3)			0.87 (*dd*, *J*= 6.71, 2.07, 3H)	22.38 (CH_3)		
	**Phenylalanine**		**Phenylalanine**		**Valine**		**Phenylalanine**	
1′	-	167.88 (C)	-	164.52 (C)	-	167.15 (C)		165.02 (C)
2′	3.84 (*dt*, *J* = 9.7, 5.1 Hz, 1H)	58.87 (CH)	4.35 (*t*, *J* = 4.9 Hz, 1H)	56.00 (CH)	3.62 (*tt*, *J* = 2.76, 1.25, 1H)	59.9 (CH)	4.35 (*t*, *J* = 5.2 Hz, 1H)	55.36 (CH)
3′	8.03 (*d*, *J* = 4.6 Hz, 1H)	- (NH)	8.04 (*d*, *J* = 2.5 Hz, 1H)	- (NH)	8.04 (*d*, *J* = 2.68, 1H)	- (NH)	7.97 (*s*, 1H)	- (NH)
4′	3.12 (*dd*, *J* = 10.2, 7.5 Hz, 2H)	41.79 (CH_2)	3.13 – 2.99 (*m*, 1H)	35.85 (CH_2)	2.11 (*td*, *J*= 6.96, 4.08, 1H)	31.97 (CH)	3.05 (*dd*, *J* = 7.4, 5.1 Hz, 2H)	35.15 (CH_2)
5′	-	138.44 (C)	-	136.28	0.95 (*d*, *J*= 7.03, 3H) (C)	19.47 (CH_3)	-	137.21 (C)
6′	7.26 – 7.23 (*m*, 5H)	128.78 (CH)	7.36 – 7.10 (*m*, 5H)	128.38 (CH)	0.86 (*d*, *J*= 6.71, 2.07, 3H)	17.92 (CH_3)	7.23 (*dd*, *J* = 6.1, 5.0 Hz, 5H)	129.75 (CH)
7′		126.75 (CH)		126.77 (CH)				127.68 (CH)
8′		130.27 (CH)		130.16 (CH)				126.69 (CH)
9′		126.75 (CH)		126.77 (CH)				127.68 (CH)
10′		128.78 (CH)		128.38 (CH)				129.75 (CH)

Figure S1: Structures of the discriminating dereplicated metabolites detected from the OPLS-DA plot of sub-fractions listed in table 3.

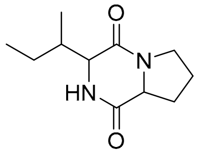

Cyclo (isoleucyl-prolyl)

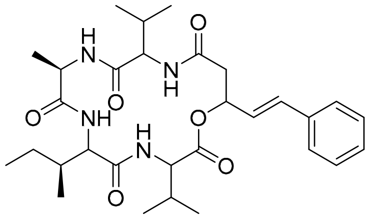

Turnagainolide A

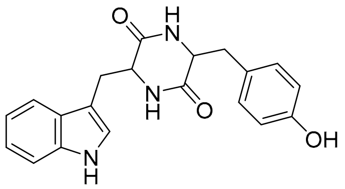

Cyclo (tryptophanyl-tyrosyl)

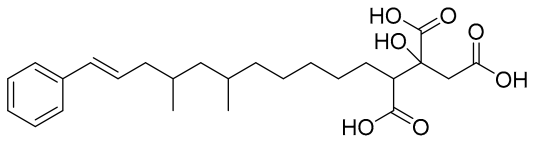

Antibiotic L 731128

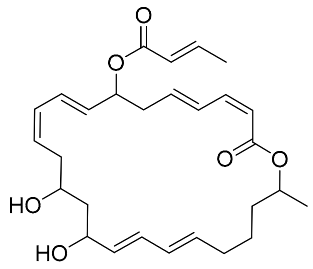

7-O-(2E-butenoyl) Macrolactin A

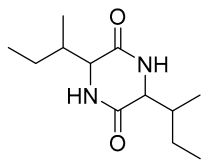

Cyclo (isoleucyl-isoleucyl)

Figure S2A: 2D correlations (COSY and HMBC) of compounds **2-5**.

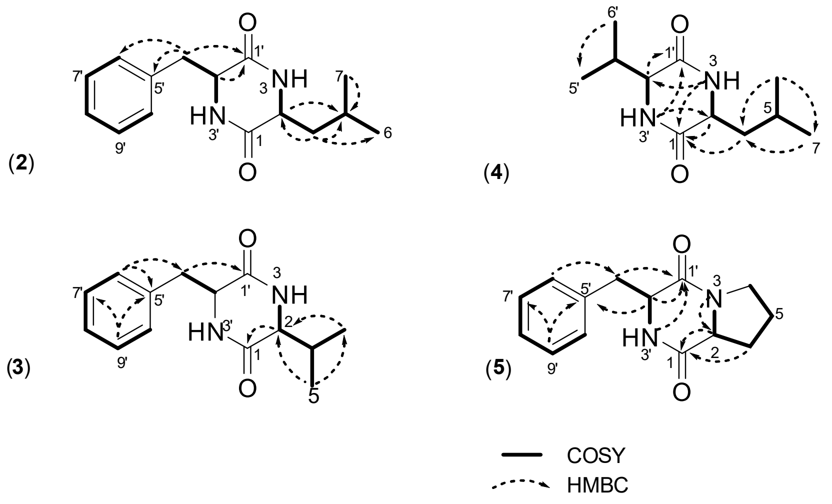

Figure S2B: ECD data of compounds **2-5** in methanol.

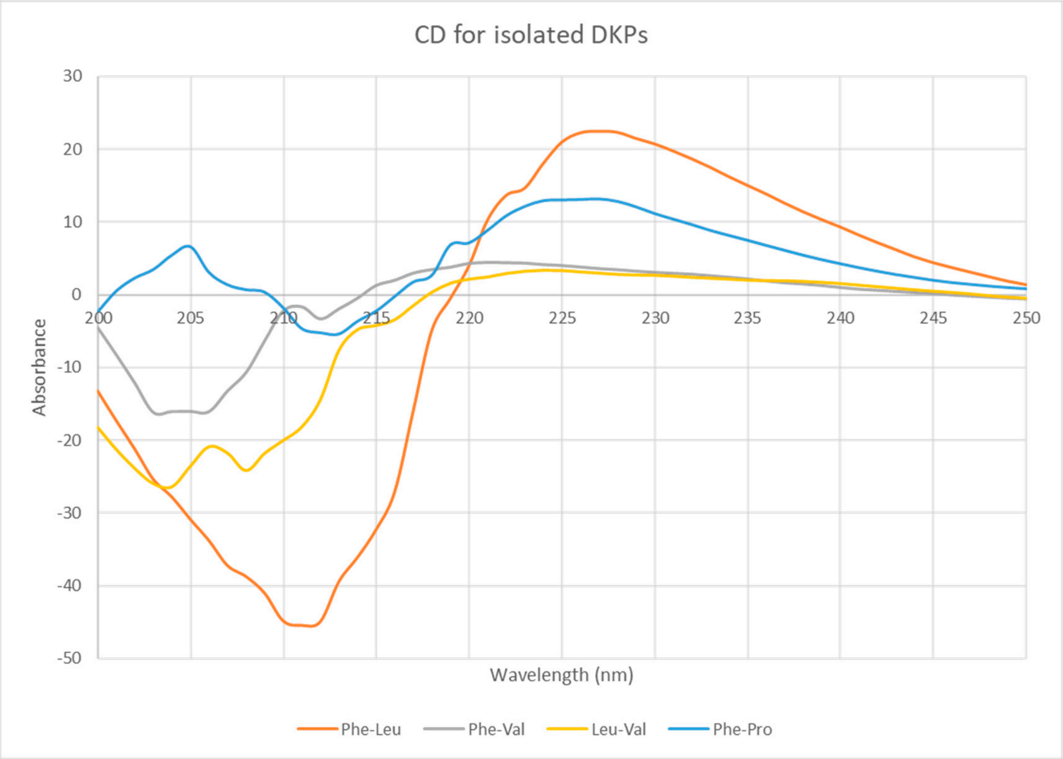

Figure S3: ^{1}H NMR spectrum of **1**

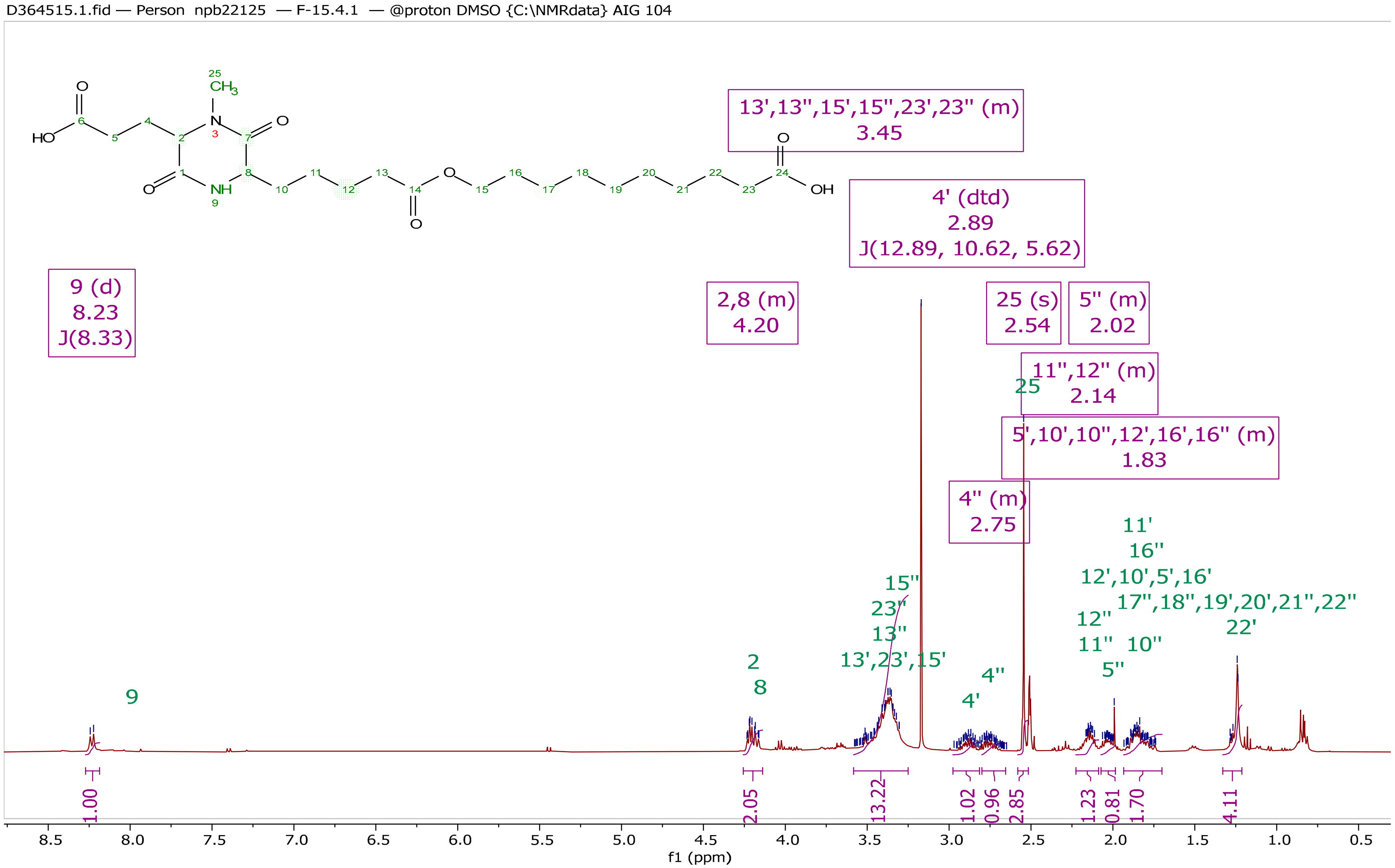

Figure S4: J Mod ^{13}C NMR spectrum of **1**

D364515.6.fid — Person npb22125 — F-15.4.1 — @DEPTQ dimethylsulfoxide-d6 {C:\NMRdata} AIG 104

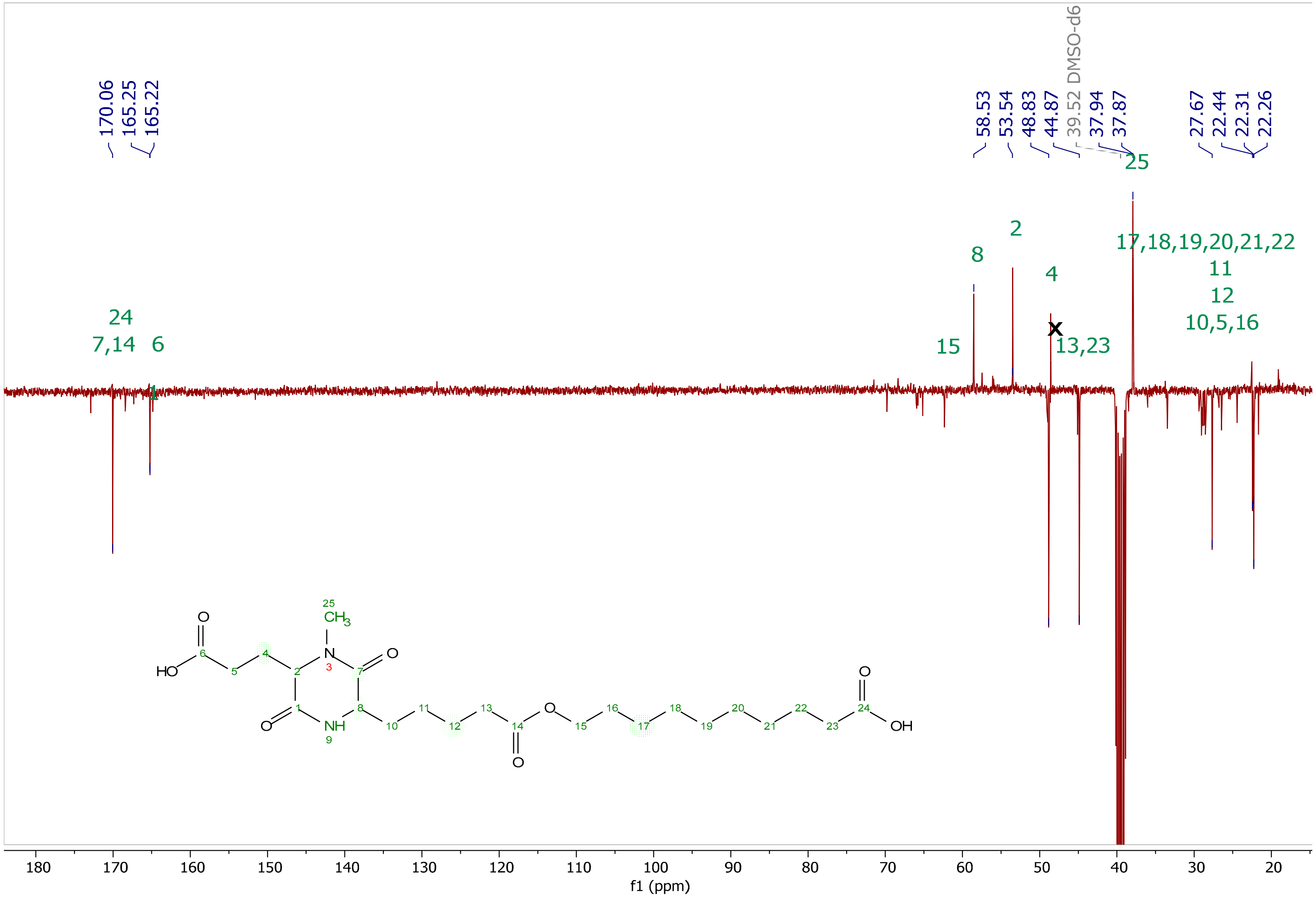

Figure S5: HMBC spectrum of **1**

D364515.4.ser — Person npb22125 — F-15.4.1 — @HMBC DMSO {C:\NMRdata} AIG 104

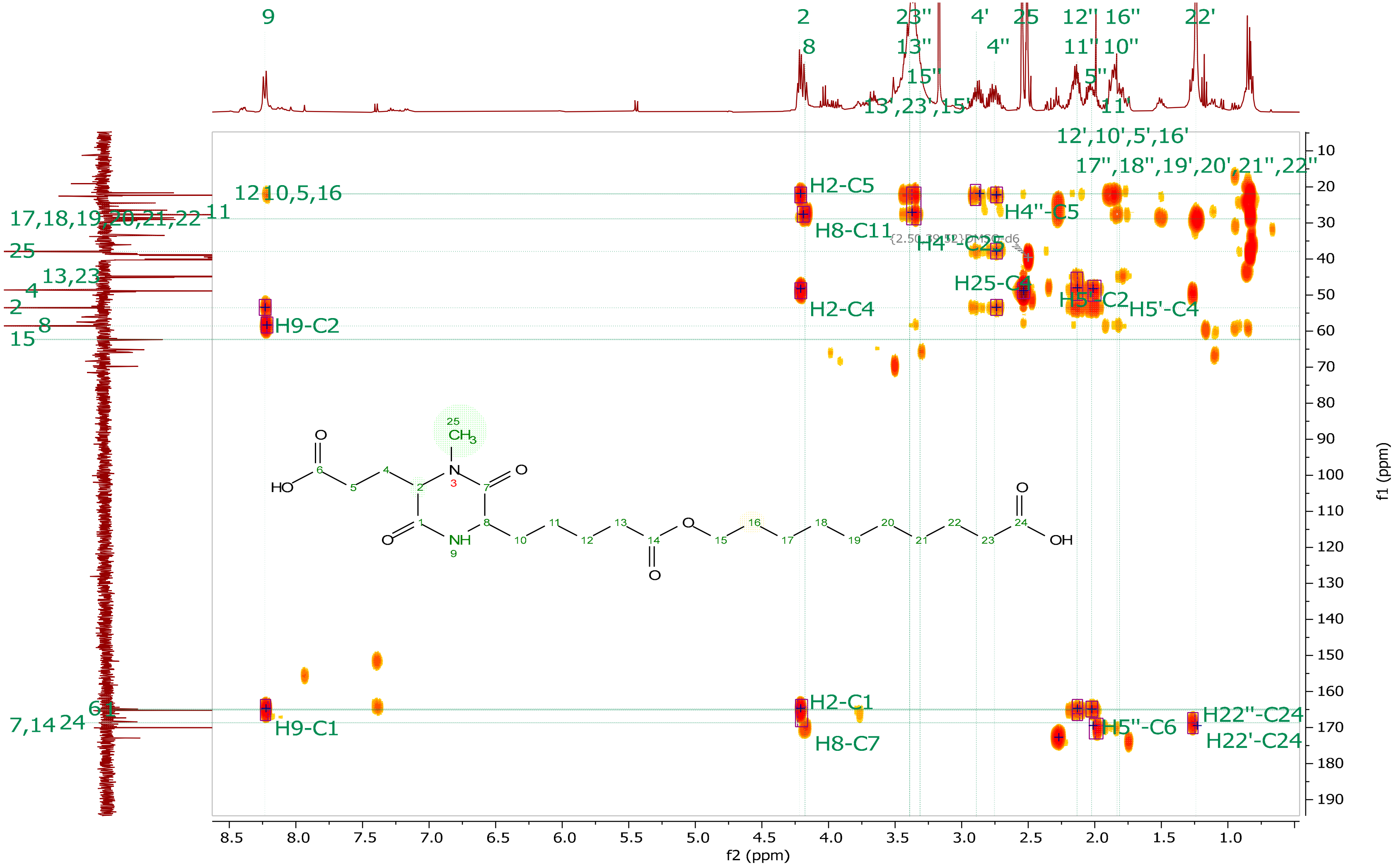

Figure S6: COSY spectrum of **1**

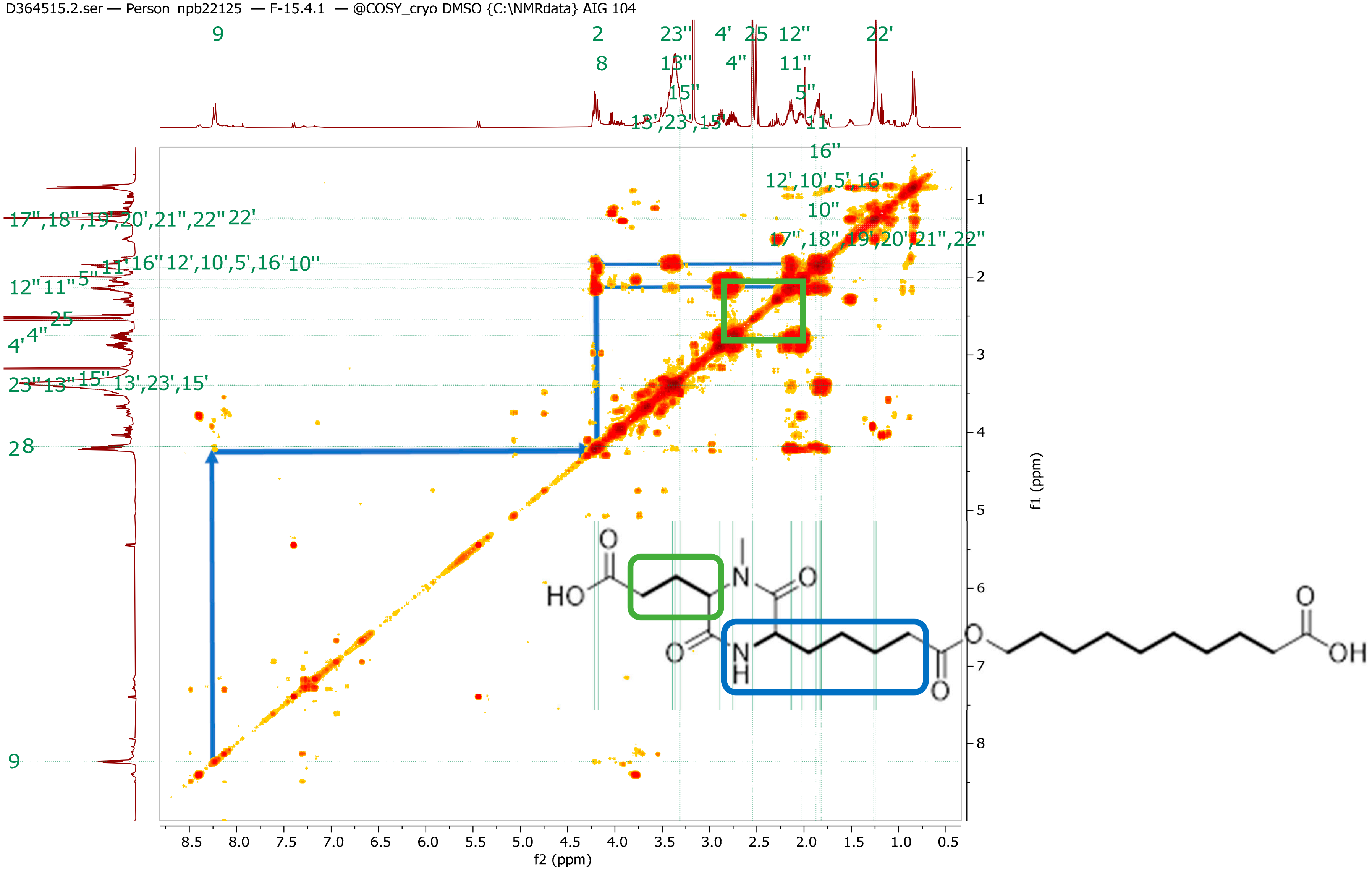

Figure S7: HSQC spectrum of **1**

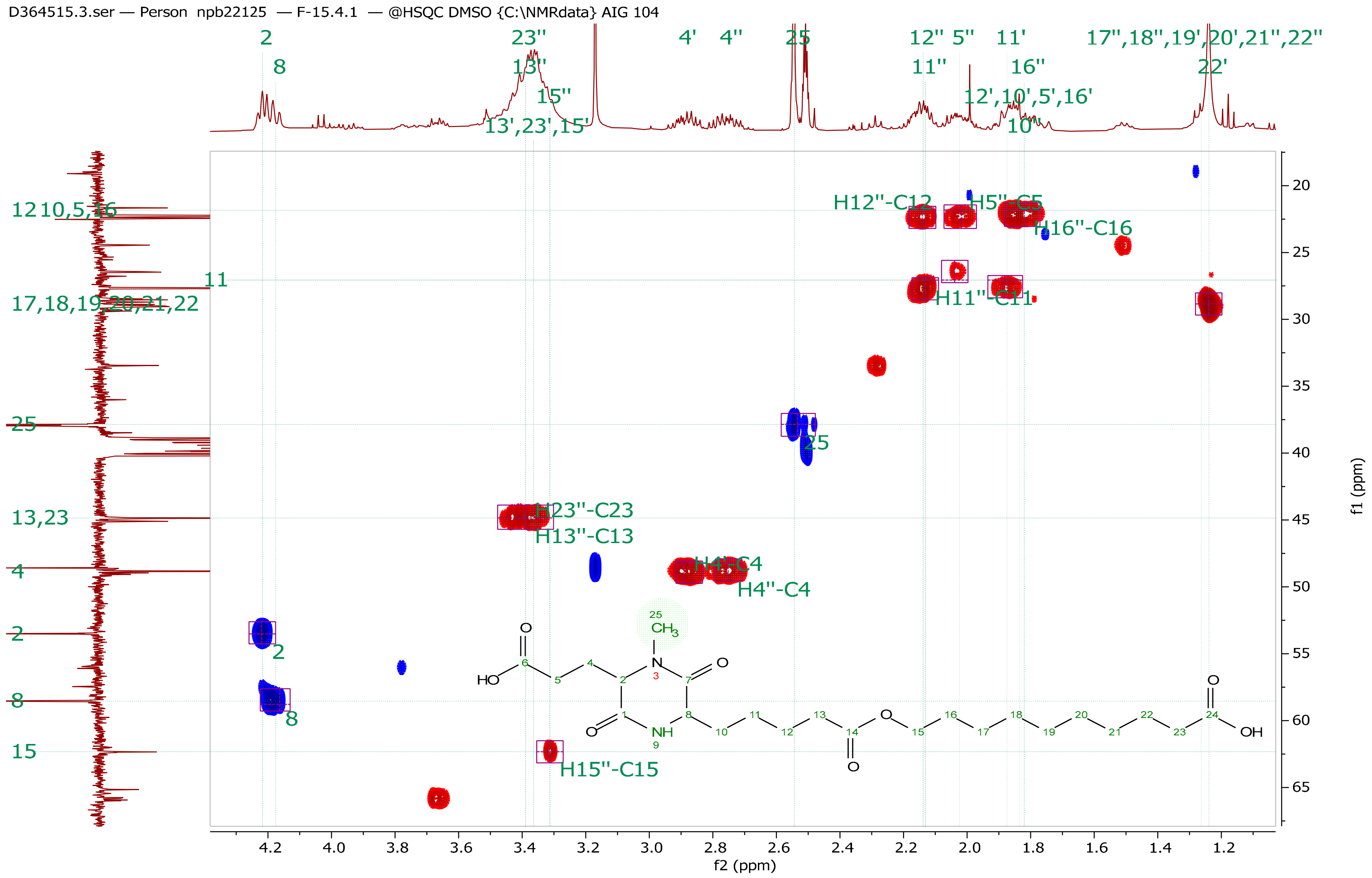

Figure S8: LC-HRMS and MS/MS fragmentation of **1**

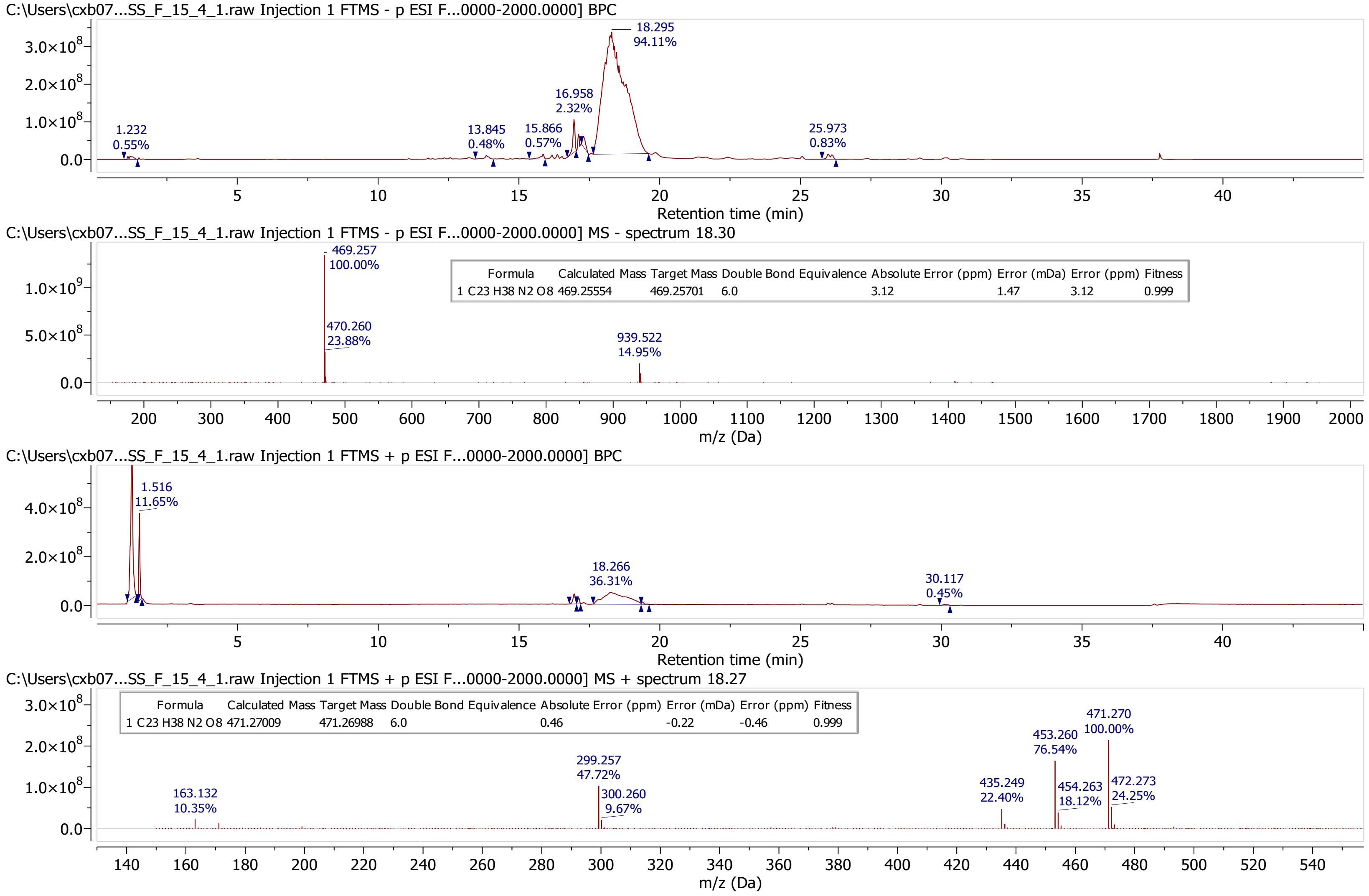

Figure S9: ECD spectrum of compound **1** in methanol.

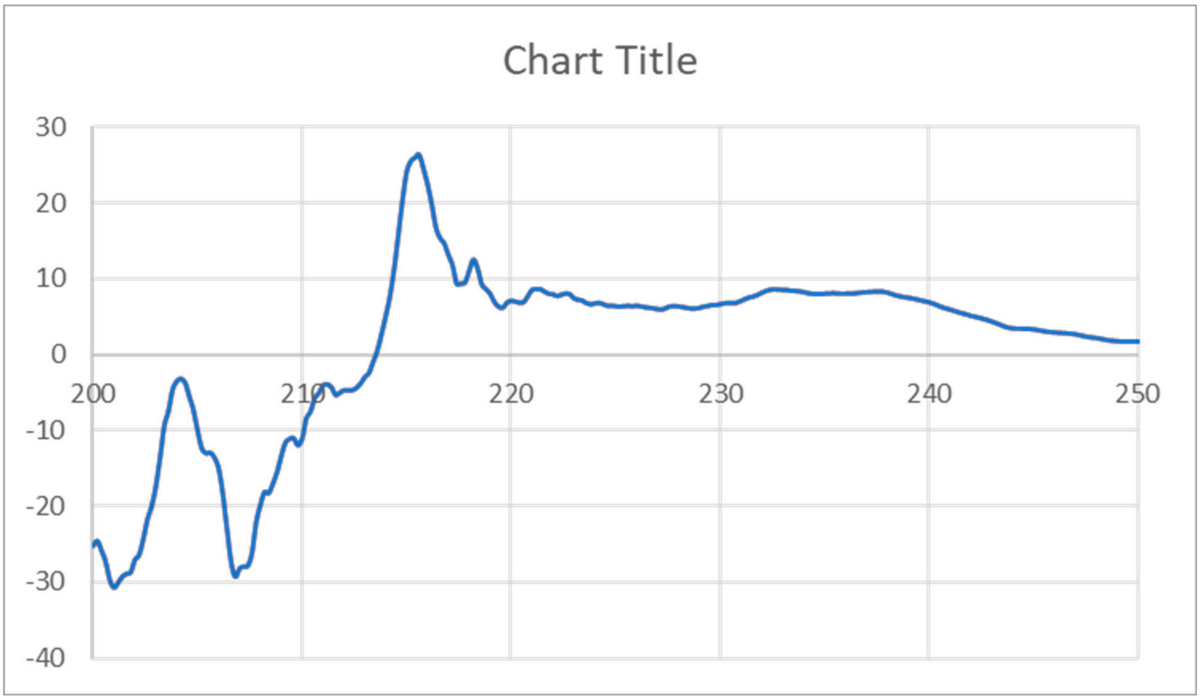

Figure S10: Post-biofilm MBEC determination of Ciprofloxacin antibiotic against *MRSA* ATCC 29213.

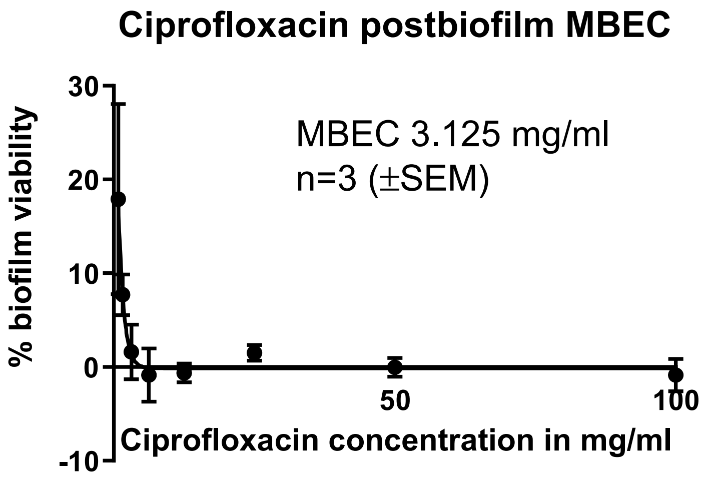

Supplement: Supplementary file 1 [file microorganisms-12-00413-s001.zip › microorganisms-2849418-supplementary.pdf]
